# 540. Impact on Clinical Decision Making of Microbial Broad Range Metagenomic Cell-Free DNA at a Single Academic Medical Center, a Retrospective Study

**DOI:** 10.1093/ofid/ofac492.593

**Published:** 2022-12-15

**Authors:** Michael C Olthoff, Takaaki Kobayashi, Meredith Parsons, Bradley A Ford, Kunatum Prasidthrathsint, Lemuel R Non, Daniel Diekema, Dilek Ince, Jorge Salinas

**Affiliations:** University of Iowa Hospitals and Clinics, Iowa City, Iowa; University of Iowa Hospitals and Clinics, Iowa City, Iowa; University of Iowa Hospitals and Clinics, Iowa City, Iowa; University of Iowa Hospitals and Clinics, Iowa City, Iowa; University of Iowa Hospitals and Clinics, Iowa City, Iowa; University of Iowa Hospitals and Clinics, Iowa City, Iowa; University of Iowa Hospitals and Clinics, Iowa City, Iowa; University of Iowa Hospitals and Clinics, Iowa City, Iowa; University of Iowa Hospitals and Clinics, Iowa City, Iowa

## Abstract

**Background:**

Broad-range metagenomic cell-free DNA testing (Karius®) can identify a variety of pathogens from a single blood sample to help in diagnosis of deep seated and bloodstream infections. Few studies have evaluated the impact of Karius testing on clinical decision making when compared to more conventional diagnostic methods.

**Methods:**

We performed a retrospective cohort study of adult patients who had Karius tests at University of Iowa Hospitals and Clinics between 01/2020 and 10/2021. Chart review was conducted to obtain patient characteristics and clinical course including patient age, immunocompromising conditions, involvement of Infectious Disease (ID) consultant, reason for the test, and final diagnosis. Results of the Karius tests and conventional tests were compared for concordance. Clinical impact and change in management due to Karius testing were determined using previously established criteria outlined by Hogan et al.(Clin Infect Dis. 2021 Jan 27;72(2):239-245)

**Figure:**
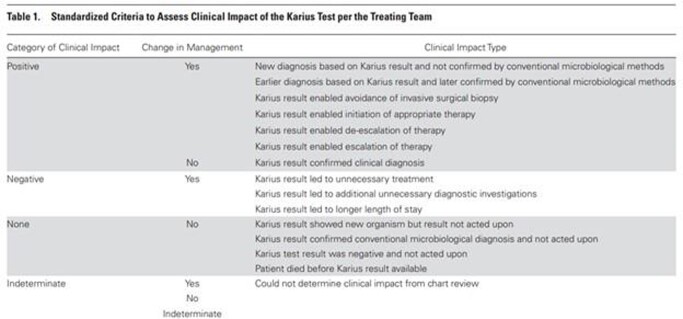

**Results:**

Out of 26 patients who had Karius testing, 20 patients (77%) had an immunocompromising condition, the most common of which was hematologic malignancy (17 patients, 65%). Twenty-four patients (92%) had received a formal ID consultation prior to the test. The most common finding prior to testing was the presence of pulmonary infiltrates on imaging (20 patients, 77%). Karius testing was positive in 18/26 cases (69%). Organisms detected by Karius and conventional testing were concordant in 9/18 cases (50%). Karius test result had a positive clinical impact in 3 cases (12%), an indeterminant impact in 1 case (3%) and no impact in 22 cases (85%). Reasons for a positive impact are as follows: 1 case with initiation of antituberculosis therapy due to earlier diagnosis of tuberculosis, 1 case with de-escalation of antifungal therapy based on negative Karius results, and 1 case of clearance for urgent lung transplantation based on negative Karius results in the setting of mobile echo density on echocardiogram in the absence of other stigmata of endocarditis.

**Figure:**
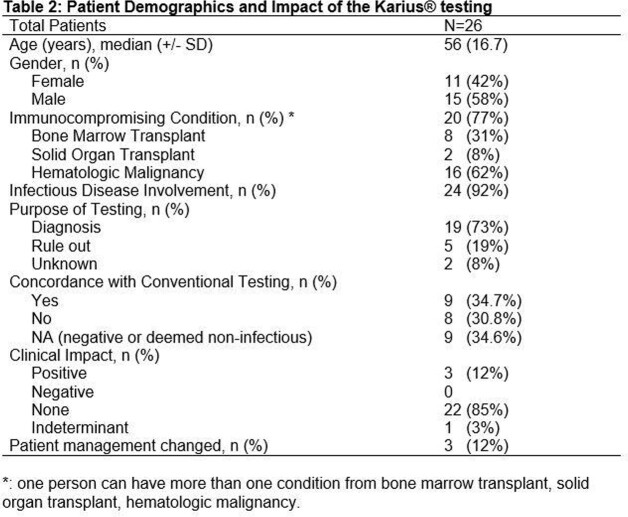

**Conclusion:**

The impact of Karius testing on patient management appears limited. Further studies will be needed to identify patient populations or disease factors in which this testing have clinical impact.

**Disclosures:**

**All Authors**: No reported disclosures.

